# Medical Correctness and User Friendliness of Available Apps for Cardiopulmonary Resuscitation: Systematic Search Combined With Guideline Adherence and Usability Evaluation

**DOI:** 10.2196/mhealth.9651

**Published:** 2018-11-06

**Authors:** Bibiana Metelmann, Camilla Metelmann, Louisa Schuffert, Klaus Hahnenkamp, Peter Brinkrolf

**Affiliations:** 1 Department of Anaesthesiology University Medicine Greifswald Greifswald Germany

**Keywords:** mHealth, resuscitation, review, guidelines, mobile phones, health care information systems, health informatics

## Abstract

**Background:**

In case of a cardiac arrest, start of cardiopulmonary resuscitation by a bystander before the arrival of the emergency personnel increases the probability of survival. However, the steps of high-quality resuscitation are not known by every bystander or might be forgotten in this complex and time-critical situation. Mobile phone apps offering real-time step-by-step instructions might be a valuable source of information.

**Objective:**

The aim of this study was to examine mobile phone apps offering real-time instructions in German or English in case of a cardiac arrest, to evaluate their adherence to current resuscitation guidelines, and to test their usability.

**Methods:**

Our 3-step approach combines a systematic review of currently available apps guiding a medical layperson through a resuscitation situation, an adherence testing to medical guidelines, and a usability evaluation of the determined apps. The systematic review followed an adapted preferred reporting items for systematic reviews and meta-analyses flow diagram, the guideline adherence was tested by applying a conformity checklist, and the usability was evaluated by a group of mobile phone frequent users and emergency physicians with the system usability scale (SUS) tool.

**Results:**

The structured search in Google Play Store and Apple App Store resulted in 3890 hits. After removing redundant ones, 2640 hits were checked for fulfilling the inclusion criteria. As a result, 34 apps meeting all inclusion criteria were identified. These included apps were analyzed to determine medical accuracy as defined by the European Resuscitation Council’s guidelines. Only 5 out of 34 apps (15%, 5/34) fulfilled all criteria chosen to determine guideline adherence. All other apps provided no or wrong information on at least one relevant topic. The usability of 3 apps was evaluated by 10 mobile phone frequent users and 9 emergency physicians. Of these 3 apps, solely the app “HELP Notfall” (median=87.5) was ranked with an SUS score above the published average of 68. This app was rated significantly superior to “HAMBURG SCHOCKT” (median=55; asymptotic Wilcoxon test: z=−3.63, *P*<.01, n=19) and “Mein DRK” (median=32.5; asymptotic Wilcoxon test: z=−3.83, *P*<.01, n=19).

**Conclusions:**

Implementing a systematic quality control for health-related apps should be enforced to ensure that all products provide medically accurate content and sufficient usability in complex situations. This is of exceptional importance for apps dealing with the treatment of life-threatening events such as cardiac arrest.

## Introduction

### Background

In case of a cardiac arrest, cardiopulmonary resuscitation (CPR) has to start as soon as possible [1]. However, even in the most advanced emergency systems, the emergency personnel needs a medium time of 5-8 min to arrive at the emergency site [2]. Therefore, it is crucial that a bystander—that is, a person noticing the cardiac arrest—starts CPR [3,4]. In the majority of cases, the bystander will be a medical layperson [5]. It has been shown in multiple studies that bystander CPR increases the rate of survival [1,6-9]. Nevertheless, the rate of bystander CPR is still relatively low [10]. Various reasons for this gap are discussed [11,12]. One of them might be that the bystander has probably never experienced a similar situation before and is therefore unsure what to do and fears to make mistakes [13]. The situation being highly time-critical further increases the cognitive workload. Decisions have to be made fast, leaving no time for elaborate reflections. It has been shown that cognitive aids can help reduce stress in these types of situations [14]. However, because cardiac arrests can happen anytime and at any place, it is unlikely that the bystander carries a traditional cognitive aid such as a textbook or leaflet with the required information.

### Objectives

A possible solution to allow immediate assistance might be a mobile phone app offering real-time step-by-step instructions. Mobile electronic devices such as mobile phones are ubiquitously available and a bystander is likely to have them available at site [15]. Mobile phone apps have become a part of everyday culture and have changed daily life in nearly all aspects [16]. Especially digital natives are used to being able to receive information immediately; internet research and apps are their first choice in cases of questions and often the main source of information [16,17]. Likewise, the market of mHealth apps has grown exponentially over the last years [18-20]. Cognitive aids, which are based on medical guidelines, are increasingly accepted in health care [21]. Ahn et al described that the total number of downloads for CPR training apps is about several hundred thousand [22]. However, this field is getting increasingly complex and unmanageable [23]. Kumar et al raised concerns toward untested apps already at the mHealth Evidence Workshop at the US National Institutes of Health in 2011 [24]. They demanded rigorous research to examine the potentially negative consequences of ineffective mHealth apps or apps based on incorrect facts. This could lead to patient harm and higher medical costs [24]. It cannot be expected from medical laypersons to analyze all possible apps, evaluate the content, and decide whether it conforms to current medical guidelines.

The aim of this study was to systematically detect apps giving German or English step-by-step instructions to perform CPR by an adult bystander and determine both their adherence to current medical guidelines and their usability.

## Methods

### Study Setup

Our 3-step approach combined (1) a systematic review of currently available apps guiding a medical layperson through a resuscitation situation, (2) an adherence testing to medical guidelines, and (3) a usability evaluation of the determined apps. The study was approved by the institutional review board of Universitätsmedizin Greifswald with the case number BB 055/17.

### Systematic Review of Available Apps

To date, there is no standardized search method for identifying mobile health apps. We used an approach similar to other studies [25-27]. The search was structured to an adapted preferred reporting items for systematic reviews and meta-analyses (PRISMA) flow diagram [28].

We focused on the 2 largest and most popular stores for mobile apps—Apple App Store (for Apple iOS apps) and the Google Play Store (for Android apps). Smaller stores such as Amazon App store, Windows Store, Samsung Apps, or Blackberry World were not included in this study [29-31]. The apps offered in these 2 app stores are automatically preselected depending on the region from where the search is conducted. Google Play Store identifies the location based on the users’ IP address, whereas Apple App Store uses Apple ID. This default country setting can be changed [32,33]. An extensive search with country settings of all English-speaking countries would have led to an unmanageable amount of apps; whereas a restriction to just a few selected English-speaking countries would have been arbitrary. Therefore, we decided to restrict the search to the country setting of Germany. We defined 16 keywords and hand-searched each term separately. These keywords were the English words “CPR,” “resuscitation,” “chest compression,” “basic life support,” “BLS,” “first aid,” “cardiac arrest,” “112,” and similar German expressions (“Reanimation,” “Wiederbelebung,” “Thoraxkompression,” “Herzdruckmassage,” “Erste Hilfe,” “Herzstillstand,” “Kreislaufstillstand,” and “Notfall”). The systematic search was carried out on a MacBook Pro between May 26, 2017, and June 23, 2017. For Apple App Store, the iTunes search configuration was set to “all” (Mac, iPad, iPhone, and Apple Watch). Google Play Store was searched on the same MacBook Pro [34] accessed via Safari internet browser. Consistent with other studies, apps were identified if the keyword was either part of the title or the description of the app [27].

All identified apps were screened. Apps that were found under different keywords but had the same name and were developed by the same company were considered redundant.

For assessment of eligibility, the remaining apps were examined regarding the study’s inclusion criteria. Apps not coherent with 1 or more of these criteria were excluded from further evaluation. The following inclusion criteria were used in a descending rank order: availability on both Google Play Store and Apple App Store, language of the app either German or English, free of charge, covered the topic resuscitation, resuscitation of human beings, provides real-time step-by-step instructions, no duplicate under different names, and no technical problems. The inclusion criterion “availability in both stores” was chosen to find an app that can be recommended in, for example, basic life support trainings and is usable by the majority of mobile phone users without being restricted to a subgroup. Apps were classified as duplicates if they were developed by the same company and had the same interface but had different names. All remaining apps were included for evaluation of guideline adherence.

### Conformity to Guideline

We analyzed the quality of content based on the adherence to the European Resuscitation Council Guidelines for Resuscitation 2015 [2], the American Heart Association Guidelines Update for Cardiopulmonary Resuscitation and Emergency Cardiovascular Care 2015 [35], and the 2015 International Consensus on Cardiopulmonary Resuscitation and Emergency Cardiovascular Care Science With Treatment Recommendations guidelines of the International Liaison Committee on Resuscitation [36,37]. A conformity checklist was developed in 2 successive brainstorming sessions of 8 emergency physicians and paramedics containing the following 9 items: the app should request the user to “check responsiveness,” “open the airway,” “assess whether the person is breathing normally (see, hear, and feel),” “consider no breathing or abnormal, ie, agonal breathing,” “call 112 (or 911) or ask somebody to call 112 (or 911),” “start chest compression,” “pay attention to correct positioning of hands,” “compress the chest at a rate of 100-120 bpm,” and “compress to a depth of at least 5 cm but not more than 6 cm.” If the app explained ventilation, it was expected to include “opening of the airway” and “verify rising of the chest.” To be rated as guideline-conform, all criteria needed to be covered in substance; verbatim coverage was not required.

### Usability Evaluation

Remaining apps were evaluated using the system usability scale (SUS) developed by John Brooke [38]. This tool is based on the 3 categories of International Organization for Standardization norm 9241-11 for usability: “effectiveness,” “efficiency,” and “satisfaction” [38,39]. The SUS was rated as a highly robust and versatile tool to evaluate usability [40]. It is the most widely used scale to evaluate usability and has been cited in more than 600 research publications [41]. The questionnaire of the SUS consists of 10 statements on a 5-point Likert scale with 5 positive statements (item 1, 3, 5, 7, and 9) and 5 negative statements (item 2, 4, 6, 8, and 10). The user is asked to rate their level of agreement with these statements concerning the software under review. To get a total score between 0 and 100, the individual scores are calculated as follows: each item’s score ranges from 1 to 5 depending on the position. In case of the uneven items, the scale position minus 1 contributes to the total score. In case of the even items, the contribution to the total score is 5 minus the scale position. In the next step, the sum of these results is multiplied by 2.5. This results in a value on a scale between 0 and 100 [38]. This value does not represent a percentage of usability [42]. The German version of the SUS used is attached in Multimedia Appendix 1. When translating the SUS questionnaire from its original version, we changed the general term “system” into the more specific word “app.”

Sauro and Lewis detected a rate of 11% of coding errors when working with a conventional SUS score with positive and negative statements [43]. Therefore, in our evaluation, 2 researchers calculated the SUS score independently to diminish the rate of mistakes in calculation. If their results did not match, a third researcher calculated the SUS score.

#### Usability Evaluators

Barnum recommends asking multiple groups to evaluate with SUS to emphasize different aspects of a given system [44]. Regarding this topic, 3 groups, whose SUS evaluation could show different perspectives, were identified: (1) people with a higher chance of having to use the app, (2) experienced app users, and (3) people with high experience regarding the medical content of the app. The highest risk of being confronted with cardiac arrest can be attributed to elderly people [45] as well as professionals of the medical field. However, the percentage of individuals owning a mobile phone is by far smaller among people aged 65 years and older than found in the average population [46]. People working in medical environments are educated and trained in basic life support and unlikely to need the help of an app to perform the basic steps of resuscitation. Thus, we decided not to interview these groups and focus on the remaining 2: those who frequently use related products (apps) and those whose work is relevant to the content of the product. Consequently, 1 group evaluating the app consisted of mobile phone frequent users, whereas the other group consisted of emergency physicians. Mobile phone frequent users were defined as individuals who had owned a mobile phone for more than 3 years, currently have more than 15 apps installed on their device, and use these for more than 1 hour per day. The emergency physicians are all currently employed in the German emergency system. The emergency physicians were asked to keep in mind that the apps were designed to teach the steps of basic life support to medical laypersons.

In addition, the emergency physicians were asked to rank the apps according to the quality of teaching different aspects of high-quality CPR: 7 aspects were developed in 2 successive brainstorming sessions of 8 emergency physicians and paramedics based on the German translation of the European Resuscitation Council guidelines. Aspects, all researchers involved associated with high quality CPR were collected and evaluated. The criteria were as follows: “The app comprehensively explains the opening of the airway,” “The app points out the problem of agonal breathing,” “The app emphasizes the importance of complete recoil of the chest after each compression,” “The app indicates that pauses in chest compressions should be minimized,” “The app helps the medical layperson to find the correct frequency for chest compression,” “The design and user-interface supports an optimal execution of cardiopulmonary resuscitation,” and “The app requests the medical layperson to continue with cardiopulmonary resuscitation until the arrival of emergency service.”

Statistical processing of the data was carried out using IBM SPSS Statistics, version 26.0 (IBM Corporation, Armonk, New York, USA), and Microsoft Excel 2010 (Microsoft Corporation, Redmond, Washington, USA). We assessed normal distribution by the Shapiro-Wilk test; median and interquartile range were calculated. In case of normal distribution, *t* test was used to assess significance levels. To assess significance level in the absence of normal distribution, Mann-Whitney *U* test was used between the 2 groups testing the same app and Wilcoxon test between the same people testing different apps.

## Results

### Systematic Review of Apps

**Figure 1:**
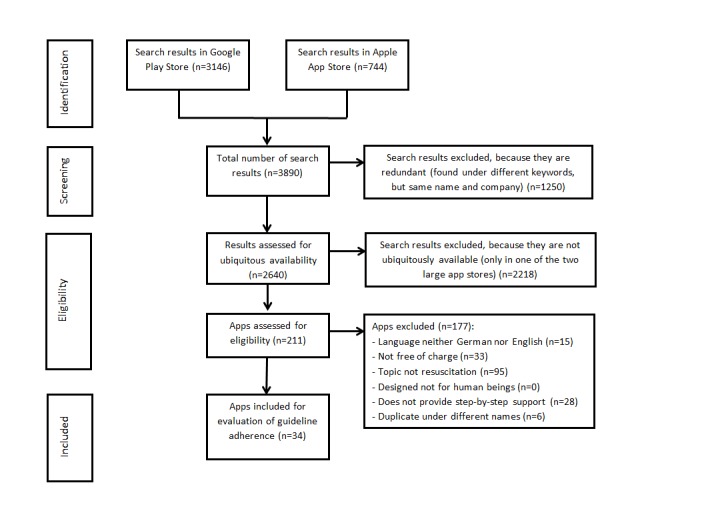
Results of the systematic review of apps providing step-by-step instructions for cardiopulmonary resuscitation (CPR) in case of a cardiac arrest.

The results of the systematic review are depicted in Figure 1 as an adapted PRISMA flow diagram. The search of the 16 German and English keywords identified 3146 search results in Google Play Store and 744 in Apple App Store. After the exclusion process, 34 apps remained for the evaluation of guideline adherence (see Multimedia Appendix 2).

### Adherence to Guideline

**Figure 2:**
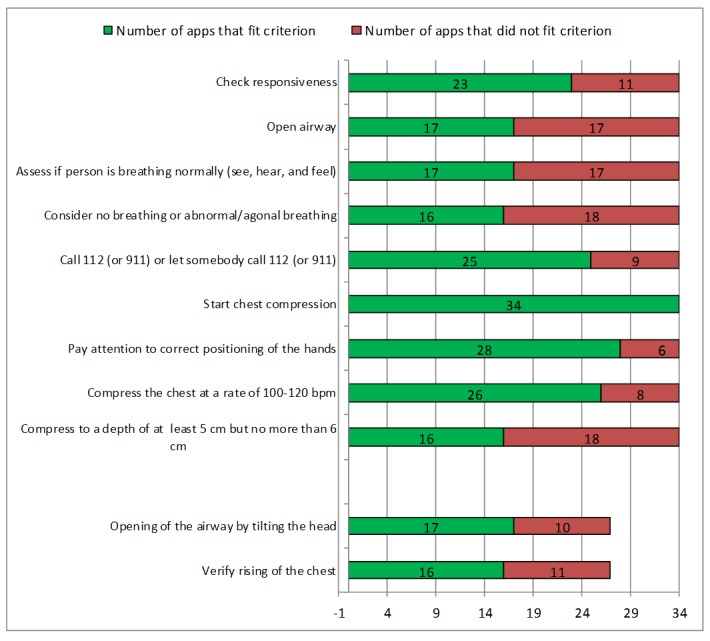
Apps in accordance with the criteria used to evaluate guideline adherence.

The results of the analysis of guideline adherence are depicted in Figure 2. A total of 7 apps taught hands-only CPR, whereas 27 also explained ventilation. Out of the 34 apps analyzed, 18 (53%, 15/34) did not indicate to consider “no breathing and abnormal, ie, agonal breathing,” 17 (50%, 17/34) did not request to assess whether the person is breathing normally, 18 (53%, 18/34) did not explain to compress to the recommended depth of at least 5 cm but not more than 6 cm, and 17 (50%, 17/34) did not recommend to open the airway. In our evaluation, only 5 out of the 34 (15%, 5/34) apps met all criteria of guideline adherence tested; these apps are listed in Table 1.

### Usability Evaluation

The usability evaluation with the SUS tool was conducted in October 2017. Of the 5 apps that met the criteria, 2 had to be excluded before starting the usability evaluation: 1 app was no longer available in both app stores (“St John Wales First Aid”) and the other app (“Notfall-Hilfe”) was excluded because it showed fundamental differences between the version of the Google Play Store and Apple App Store. The version of the Google Play Store contained pictures and movies explaining all steps, and the text was read aloud, if desired. None of these features were available in the Apple App Store version. This gap would profoundly influence the results of the SUS, leading to the decision to exclude this app in the usability evaluation.

The group of mobile phone frequent users consisted of 10 participants (7 females and 3 males) with a median age of 23 years (minimum=20 years and maximum=25 years) and the group of emergency physicians of 9 participants (4 females and 5 males) with a median age of 37 years (minimum=32 years and maximum=56 years). The SUS participants assessed the apps on their own mobile phone. iPhone 6Plus, provided by the researchers, was used by 2 emergency physicians.

**Table 1:** Apps that met all 9 of our criteria for guideline adherence.

Name	Company	Google Play Store version number	Apple App Store version number
HELP Notfall	Schweizerische Herzstiftung	1.0	1.0
HAMBURG SCHOCKT	Arbeiter-Samariter-Bund Hamburg	1.5.0	1.5.0
Mein DRK-Die Rotkreuz-App des DRK e.V	Deutsches Rotes Kreuz e.V	2.5.5	2.8.3
St John Wales First Aid	St John Cymru Wales	1.03	Unknown
Notfall-Hilfe	PASS Consulting Group	4.0	3.9.3

**Figure 3:**
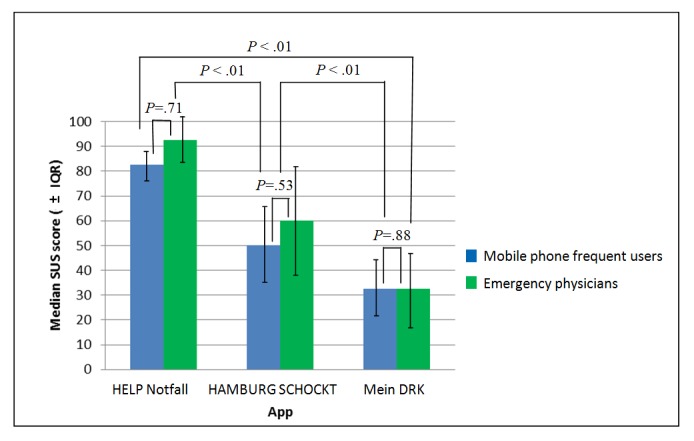
Usability evaluation of the apps with system usability scale (SUS) score. IQR: interquartile range.

The median SUS of “HELP Notfall” was significantly higher than “HAMBURG SCHOCKT” (87.5 vs 55; asymptotic Wilcoxon test: z=−3.63, *P*<.01, n=19) and also significantly higher than “Mein DRK” (87.5 vs 32.5; asymptotic Wilcoxon test: z=−3.83, *P*<.01, n=19). The median SUS of “HAMBURG SCHOCKT” was significantly higher than “Mein DRK” (55 vs 32.5; asymptotic Wilcoxon test: z=−2.81, *P*<.01, n=19). The median SUS scores did not differ significantly between the group of mobile phone frequent users and emergency physicians. The SUS results are shown in Figure 3.

Table 2 depicts how the emergency physicians ranked the apps according to the quality of teaching different aspects of high-quality CPR. Of the 9 emergency physicians, 1 did not complete this part of the questionnaire. The participating emergency physicians rated the app “HELP Notfall” as the one, teaching the majority of relevant aspects (6 out of 7) best. There was no clear result for the aspect “The app emphasizes the importance of complete recoil of the chest after each compression.”

**Table 2:** Number of emergency physicians rating the app listed as the one teaching a specific aspect of high-quality cardiopulmonary resuscitation best. A total of 8 emergency physicians evaluated the apps.

Specific aspects of high-quality cardiopulmonary resuscitation	HELP Notfall, n (%)	HAMBURG SCHOCKT, n (%)	Mein DRK, n (%)
The app comprehensively explains the opening of the airway	7 (88)	1 (12)	0 (0)
The app points out the problem of agonal breathing	8 (100)	0 (0)	0 (0)
The app emphasizes the importance of complete recoil of the chest after each compression	3 (38)	4 (50)	1 (12)
The app indicates that pauses in chest compressions should be minimized	7 (88)	1 (12)	0 (0)
The app helps the medical layperson to find the correct frequency for chest compression	8 (100)	0 (0)	0 (0)
The design and user interface supports an optimal execution of cardiopulmonary resuscitation	8 (100)	0 (0)	0 (0)
The app requests the medical layperson to continue with cardiopulmonary resuscitation until the arrival of emergency service	8 (100)	0 (0)	0 (0)

## Discussion

### Principal Findings

The structured search in 2 app stores resulted in 3890 hits. After removing redundant ones, 2640 hits were checked for fulfilling the inclusion criteria. Hereby, 34 apps were identified, meeting all inclusion criteria, of which only 5 (15%, 5/34) fulfilled all defined criteria of adherence to the guidelines of the European Resuscitation Council and the American Heart Association. All other apps gave no or incorrect information on at least one relevant topic. Regarding the usability, only 1 out of the 3 apps was evaluated with an SUS score above the published average of 68 [41].

#### Systematic Review of Apps

The systematic review of apps available on Google Play Store and Apple App Store took place between May 26, 2017, and June 23, 2017. After excluding redundant hits; duplicates apps; and apps that were not ubiquitously available, free of charge, or did not provide step-by-step instructions in English or German for resuscitation of human beings, 34 apps remained.

Similar to other studies, the search in Google Play Store yielded far more results than the Apple App Store [47]. One reason for this striking difference might be the different submission systems. Although there are no admission requirements in the Google Play Store, Apple tests each submitted app for technical compatibility and conducts a content verification review [27,48]. However, the main goal of this content verification seems to be to ensure that the name and description of the app match with the content.

Various aspects increase the difficulties for a medical layperson to find a suitable app: the sheer volume of apps to choose from can overwhelm the user [27]. Which app will be downloaded by the user depends on a number of factors such as user ratings, appealing of screenshots, keywords, and number of downloads [49]. Therefore, in the last years, the term “app store optimization” was coined describing strategies to increase the likelihood of an app being downloaded [49,50]. Moreover, the availability of apps differs not only between operators but also between countries, which influences and limits the user’s choice.

#### Adherence to Guidelines

Of the 34 examined apps, only 5 (15%, 5/34) apps fit all of our criteria of guideline-adherent resuscitation. This alarming result is concordant with that of other studies examining the content of mHealth apps [51-54]. The “European Resuscitation Council Guidelines for Resuscitation 2015” [55] and the “American Heart Association Guidelines Update for Cardiopulmonary Resuscitation and Emergency Cardiovascular Care 2015” [35] are well known and highly respected international medical guidelines, which are evidence based on current literature. These guidelines offer clear and easy-to-understand advice on which steps should be taken to resuscitate a person. All flowcharts, pictures, and movies are freely available and translated into many different languages [56,57]. Nevertheless, only few apps implemented these recommendations. This might possibly lead to reduced probability of survival of the victim resuscitated.

It has been shown in multiple studies [58-60] that a cardiac arrest victim showing abnormal, ie, agonal breathing has an increased chance of survival compared with cardiac arrest patients suffering from apnea. However, agonal breathing is often misjudged by medical laypersons not realizing the need for performing CPR in these cases. Therefore, it is crucial that CPR apps point out that patients presenting with agonal breathing are also in need of CPR. More than half of the examined apps (53%, 18/34) did not consider this important fact. Furthermore, 50% (17/34) of the apps did not even guide the user to open the airway and 50% (17/34) did not recommend to assess whether the person is breathing (see, hear, and feel). Of the 34 apps studied, 18 (53%, 18/34) recommended no or wrong compression depth, despite numerous studies suggesting to compress to a depth of at least 5 cm but not more than 6 cm to increase the chance of a positive medical outcome [61-65]. Of the 34 apps, 8 (24%, 8/34) apps did not show the correct compression rate of 100-120 bpm that has been proven to improve survival rates [66]. Of the 34 apps, 27 additionally explained ventilation, although the European Resuscitation Council as well as the American Heart Association recommend to teach medical laypersons hands-only CPR [35,55]. The 2 additional criteria for apps explaining ventilation as well were not met by all of these apps. Although this further diminishes the number of apps meeting all criteria, it is not the sole reason for the low adherence rate. A long conformity checklist certainly increases the risk of an app not meeting every single criterion. However, all chosen criteria for guideline adherence are important evidence-based aspects, which are taught in resuscitation courses worldwide.

Determining a global quality management for apps is complicated by different legislations in different countries and multiple concerned governmental agencies (eg, health and privacy legislation) [67,68]. Although institutions and authorities from different states suggested approaches to a quality control of apps, there is no universally accepted procedure [69-73]. However, in our opinion, all efforts to increase the medical accuracy of mHealth products should be made. The aspiration of all persons teaching CPR should be that the general public is provided with correct information on resuscitation, independent on how the information is spread (by textbooks, movie clips, leaflets, or apps).

#### Usability Evaluation

Of the 3 apps examined with regard to usability, the app “HELP Notfall” had the highest median SUS score (87.5), followed by “HAMBURG SCHOCKT” (55) and “Mein DRK” (32.5). This difference was seen in both evaluating groups (mobile phone frequent users and emergency physicians). There was no significant difference between the evaluating groups.

On the basis of a work by Sauro et al, an SUS score above 68 is rated as a value above average [41]. Such a score was solely achieved by “HELP Notfall.” The other 2 apps tested performed below average. The usability of an app is crucial for its implementation and usage. Sauro reported that products with an SUS score higher than 82 have a considerable chance of being recommended to a friend or colleague [41], which was reached by “HELP Notfall.” In his famous technology acceptance model, Davis (1986) stated that systems will only be used if they are perceived as useful [74]. If the user can see a clear advantage in comparison with their previous approach, users will utilize an app [75]. In the case of an app designed for the use in a time-critical and extremely challenging situation, it is even more important that the operation is highly intuitive. If a patient is in cardiac arrest and resuscitation becomes necessary, there is no time to first become acquainted with the software. Otherwise, there seems to be a relevant risk of apps not helping the user but leading him astray from doing best for the patient. Thus, a high usability of the app is crucial.

### Limitations

The systematic review of apps was conducted in the 2 main app stores Apple App Store and Google Play Store, whereas smaller stores such as Amazon App Store, Windows Store, Samsung Apps, or Blackberry World were not included. The review was completed by only 1 researcher (LS), leaving a possibility of misjudging inclusion or exclusion criteria. However, the criteria were phrased distinctly and clearly to diminish this risk, and 2 other researchers (BM and CM) made spot checks. If an app was available either in Google Play Store or in Apple App Store but not in both stores, it was excluded because a recommendable CPR app should be usable by the majority of the population. This criterion led to the exclusion of 2218 search results, which was the majority of all search hits. Furthermore, we decided to choose “free of charge” as an inclusion criterion to enlarge the group of possible future users. A study by Lim et al conducted in different countries worldwide showed that the most important factor influencing people in the process of downloading an app was the price of the app and that 57% of users will not download apps they have to pay for [76]. We do not know how many apps explain the topic of resuscitation according to the medical guidelines in a user-friendly way but are not free of charge and have to be purchased or are available in just 1 app store. These inclusion criteria certainly influenced the amount and choice of apps analyzed. Furthermore, the world of mobile phone apps is fast moving with new apps entering the market and other ones vanishing. Hence, a review of apps always reflects availability at a certain time. We conducted the search with keywords in English and German language. We cannot say whether a search in other languages might lead to different results. The search was carried out with the app stores’ default country setting of Germany. This certainly further reduced the number of possible apps.

The SUS was evaluated by mobile phone frequent users and emergency physicians. As described in the Methods section, groups at high risk of witnessing a cardiac arrest were not included. This might bias the results.

To date, it is not known whether the use of an app providing step-by-step instructions in a CPR situation increases the rate or quality of bystander resuscitation and leads to higher survival rates among the victims. To broadly recommend the use of such apps, further studies are needed to evaluate positive and negative effects.

### Comparison With Prior Work

Only few studies systematically evaluating CPR apps exist [22,47,51-54,77-79]. In contrast to our work, some studies did not have a structured search but evaluated only a representative sample of apps [47,77] or searched only in 1 operating system [51,78]. Only a few previous studies evaluated the adherence of an app to an existing medical guideline [53,54,79]. These 3 studies covered weight loss and pain management. The study of Kalz et al included both resuscitation-teaching apps as well as apps providing guidance in a resuscitation situation in real time [52]. However, teaching a topic in a classroom and giving step-by-step instructions in a real situation are different purposes and call for different designs of the app. We decided to focus only on apps offering real-time support. This is in contrast to the study of Ahn et al, concentrating solely on CPR-training apps [22].

Contrary to all other studies, we did not select a reduced number of apps for the SUS evaluation but did a comprehensive evaluation of all apps that fit the inclusion criteria.

### Conclusions

This work combined a systematic review of currently available resuscitation apps with an assessment of guideline adherence and an evaluation of usability. The search resulted in 3890 hits. Of 34 apps that met the inclusion criteria, only 5 (15%, 5/34) fulfilled all of the criteria applied to determine guideline adherence. All other apps gave no or incorrect information on at least one relevant topic. Furthermore, our evaluation of usability revealed that only 1 of the 3 apps tested had an above average usability rate according to SUS. Implementing a systematic quality control for health-related apps should be enforced to ensure medical accuracy and sufficient usability. This is of superior importance for apps focusing on the treatment of life-threatening events such as cardiac arrest.

## Appendix

Multimedia Appendix 1 The German version of the system usability scale used.

Multimedia Appendix 2 Apps analyzed for guideline adherence.

## Abbreviations

CPR: cardiopulmonary resuscitation
PRISMA: preferred reporting items for systematic reviews and meta-analyses
SUS: system usability scale
