# Supplementary material for: Medical Correctness and User Friendliness of Available Apps for Cardiopulmonary Resuscitation: Systematic Search Combined With Guideline Adherence and Usability Evaluation
Source: JMIR Mhealth Uhealth. 2018 Nov 6;6(11):e190. doi: 10.2196/mhealth.9651 (PMC6246966; doi:10.2196/mhealth.9651)
Supplement: Multimedia Appendix 1 [file mhealth_v6i11e190_app1.pdf]

Appendix 1

Anleitung:

Bitte laden Sie sich zunächst die folgenden Applikationen auf Ihr Smartphone:

1) „HELP Notfall“ von Schweizerische Herzstiftung

AppStore: https://itunes.apple.com/de/app/help-notfall/id1076538608?mt=8

Googleplay: https://play.google.com/store/apps/details?id=com.herzstiftung.help.notfall&ddl=1&pcampaignid=web_ddl_1

2) „HAMBURG SCHOCKT“ von ASB Hamburg

AppStore: https://itunes.apple.com/de/app/hamburg-schockt/id677230620?mt=8

Googleplay: https://play.google.com/store/apps/details?id=de.HamburgSchockt

3) „MeinDRK - Die Rotkreuz-App des DRK e.V.“ von Deutsches Rotes Kreuz e.V.

AppStore: https://itunes.apple.com/de/app/meindrk-die-rotkreuz-app-des-drk-e-v/id573461478?mt=8

Googleplay: https://play.google.com/store/apps/details?id=com.adamasvision.mydrk&hl=de

Im Folgenden soll jede Applikation nach ihrer Benutzerfreundlichkeit bewerten werden. Daher sollten Sie sich vor dem Ausfüllen der Fragebögen für jede App einige Minuten Zeit nehmen und diese mit Augenmerk auf ihre Anleitung für Laien im Falle einer Wiederbelebungssituation untersuchen. Einige der Apps bieten viele weitere Funktionen. Da sich diese Studie aber mit der Anleitung der Wiederbelebung befasst, bitten wir Sie, die anderen Funktionen, insofern diese nicht auf die untersuchte Funktion Einfluss nehmen, außer Acht zu lassen.

Wenn Sie sich mit der Applikation befasst haben, bitten wir Sie den „Fragebogen 1“ für jede Applikation einzeln zu beantworten.

Bitte geben Sie zu jeder Frage an, wie Sie die entsprechende Aussage von 1- „trifft überhaupt nicht zu“ bis 5 – „trifft voll zu“ bewerten würden. Mit den dazwischen liegenden Werten können Sie Abstufungen vornehmen.

Fahren Sie erst nach dem Ausfüllen mit dem Begutachten der nächsten Applikation fort. Bitte achten Sie darauf, die Applikationen bei der Beantwortung der Fragebögen nicht zu vertauschen.

Wenn Sie für alle 5 Applikationen je einen Fragebogen ausgefüllt haben, möchten wir Sie bitten anschließend noch den „Fragebogen 2“ zu beantworten, der die Applikationen direkt miteinander vergleicht.

Dazu bilden Sie aus den von Ihnen bereits getestet Applikationen nun für jede Aussage eine Rangfolge, wobei Platz 1 mit der App zu belegen ist, die Ihrer Meinung nach die Aussage „am ehesten” erfüllt und Platz 3 „am wenigsten“ erfüllt. Bitte bilden Sie zu jeder Aussage eine Rangfolge aus allen 3 Applikationen.

Bei weiteren Fragen, können Sie sich gerne an mich wenden.

Vielen Dank für Ihre Teilnahme!

Louisa Schuffert

(Doktorandin der Klinik für Anästhesiologie, Universitätsmedizin Greifswald)

Fragebogen 1 zur System-Gebrauchstauglichkeit

„HELP Notfall“ von Schweizerische Herzstiftung

1. Ich denke, dass ich die App benutzen würde.

Stimme<br>überhaupt nicht zu<br>1	2	3	4	Stimme<br>voll zu
○	○	○	○	○

2. Ich fand die App unnötig komplex.

Stimme<br>überhaupt nicht zu<br>1	2	3	4	Stimme<br>voll zu<br>5
○	○	○	○	○

3. Ich fand die App einfach zu benutzen.

Stimme<br>überhaupt nicht zu<br>1	2	3	4	Stimme<br>voll zu<br>5
○	○	○	○	○

4. Ich glaube, ich würde die Hilfe einer fachkundigen Person benötigen, um die App benutzen zu können.

Stimme<br>überhaupt nicht zu<br>1	2	3	4	Stimme<br>voll zu<br>5
○	○	○	○	○

5. Ich fand, die verschiedenen Funktionen in dieser App waren gut integriert.

Stimme<br>überhaupt nicht zu<br>1	2	3	4	Stimme<br>voll zu<br>5
○	○	○	○	○

6. Ich denke, die App enthielt zu viele Inkonsistenzen.

Stimme<br>überhaupt nicht zu<br>1	2	3	4	Stimme<br>voll zu<br>5
○	○	○	○	○

7. Ich kann mir vorstellen, dass die meisten Menschen den Umgang mit dieser App sehr schnell lernen.

Stimme<br>überhaupt nicht zu<br>1	2	3	4	Stimme<br>voll zu<br>5
○	○	○	○	○

8. Ich fand die App sehr umständlich zu nutzen.

Stimme<br>überhaupt nicht zu<br>1	2	3	4	Stimme<br>voll zu<br>5
○	○	○	○	○

9. Ich fühlte mich bei der Benutzung der App sehr sicher.

Stimme<br>überhaupt nicht zu<br>1	2	3	4	Stimme<br>voll zu<br>5
○	○	○	○	○

10. Ich musste viel üben, bevor ich anfangen konnte die App zu verwenden.

Stimme<br>überhaupt nicht zu<br>1	2	3	4	Stimme<br>voll zu<br>5
○	○	○	○	○

Fragebogen 1 zur System-Gebrauchstauglichkeit

„HAMBURG SCHOCKT“ von ASB Hamburg

1. Ich denke, dass ich die App benutzen würde.

Stimme<br>überhaupt nicht zu<br>1	2	3	4	Stimme<br>voll zu
○	○	○	○	○

2. Ich fand die App unnötig komplex.

Stimme<br>überhaupt nicht zu<br>1	2	3	4	Stimme<br>voll zu<br>5
○	○	○	○	○

3. Ich fand die App einfach zu benutzen.

Stimme<br>überhaupt nicht zu<br>1	2	3	4	Stimme<br>voll zu<br>5
○	○	○	○	○

4. Ich glaube, ich würde die Hilfe einer fachkundigen Person benötigen, um die App benutzen zu können.

Stimme<br>überhaupt nicht zu<br>1	2	3	4	Stimme<br>voll zu<br>5
○	○	○	○	○

5. Ich fand, die verschiedenen Funktionen in dieser App waren gut integriert.

Stimme<br>überhaupt nicht zu<br>1	2	3	4	Stimme<br>voll zu<br>5
○	○	○	○	○

6. Ich denke, die App enthielt zu viele Inkonsistenzen.

Stimme<br>überhaupt nicht zu<br>1	2	3	4	Stimme<br>voll zu<br>5
○	○	○	○	○

7. Ich kann mir vorstellen, dass die meisten Menschen den Umgang mit dieser App sehr schnell lernen.

Stimme<br>überhaupt nicht zu<br>1	2	3	4	Stimme<br>voll zu<br>5
○	○	○	○	○

8. Ich fand die App sehr umständlich zu nutzen.

Stimme<br>überhaupt nicht zu<br>1	2	3	4	Stimme<br>voll zu<br>5
○	○	○	○	○

9. Ich fühlte mich bei der Benutzung der App sehr sicher.

Stimme<br>überhaupt nicht zu<br>1	2	3	4	Stimme<br>voll zu<br>5
○	○	○	○	○

10. Ich musste viel üben, bevor ich anfangen konnte die App zu verwenden.

Stimme<br>überhaupt nicht zu<br>1	2	3	4	Stimme<br>voll zu<br>5
○	○	○	○	○

Fragebogen 1 zur System-Gebrauchstauglichkeit

„MeinDRK - Die Rotkreuz-App des DRK e.V.“ von Deutsches Rotes Kreuz e.V

1. Ich denke, dass ich die App benutzen würde.

Stimme<br>überhaupt nicht zu<br>1	2	3	4	Stimme<br>voll zu
○	○	○	○	○

2. Ich fand die App unnötig komplex.

Stimme<br>überhaupt nicht zu<br>1	2	3	4	Stimme<br>voll zu<br>5
○	○	○	○	○

3. Ich fand die App einfach zu benutzen.

Stimme<br>überhaupt nicht zu<br>1	2	3	4	Stimme<br>voll zu<br>5
○	○	○	○	○

4. Ich glaube, ich würde die Hilfe einer fachkundigen Person benötigen, um die App benutzen zu können.

Stimme<br>überhaupt nicht zu<br>1	2	3	4	Stimme<br>voll zu<br>5
○	○	○	○	○

5. Ich fand, die verschiedenen Funktionen in dieser App waren gut integriert.

Stimme<br>überhaupt nicht zu<br>1	2	3	4	Stimme<br>voll zu<br>5
○	○	○	○	○

6. Ich denke, die App enthielt zu viele Inkonsistenzen.

Stimme<br>überhaupt nicht zu<br>1	2	3	4	Stimme<br>voll zu<br>5
○	○	○	○	○

7. Ich kann mir vorstellen, dass die meisten Menschen den Umgang mit dieser App sehr schnell lernen.

Stimme<br>überhaupt nicht zu<br>1	2	3	4	Stimme<br>voll zu<br>5
○	○	○	○	○

8. Ich fand die App sehr umständlich zu nutzen.

Stimme<br>überhaupt nicht zu<br>1	2	3	4	Stimme<br>voll zu<br>5
○	○	○	○	○

9. Ich fühlte mich bei der Benutzung der App sehr sicher.

Stimme<br>überhaupt nicht zu<br>1	2	3	4	Stimme<br>voll zu<br>5
○	○	○	○	○

10. Ich musste viel üben, bevor ich anfangen konnte die App zu verwenden.

Stimme<br>überhaupt nicht zu<br>1	2	3	4	Stimme<br>voll zu<br>5
○	○	○	○	○

Fragebogen 2

Bitte bilden Sie aus den von Ihnen bereits getestet Applikationen nun anhand folgender Aussagen eine Rangfolge, wobei Platz 1 mit der App zu belegen ist, die Ihrer Meinung nach die Aussage „am ehesten” erfüllt und Platz 3 „am wenigsten“. Bitte bilden Sie zu jeder Aussage eine Rangfolge aus allen 3 Applikationen.

1. Ich denke, dass ich die App benutzen würde.
    1.
    2.
    3.

2. Ich fand die App unnötig komplex.
    1.
    2.
    3.

3. Ich denke, dass die App leicht zu benutzen war.
    1.
    2.
    3.

4. Ich denke, ich würde die Unterstützung einer fachkundigen Person benötigen, um die App benutzen zu können.
    1.
    2.
    3.

5. Ich fand, die verschiedenen Funktionen der App waren gut integriert.
    1.
    2.
    3.

6. Ich halte die App für zu inkonsistent.
    1.
    2.
    3.

7. Ich glaube, dass die meisten Menschen sehr schnell lernen würden, mit der App umzugehen.
    1.
    2.
    3.

8. Ich fand die App sehr umständlich zu benutzen.
    1.
    2.
    3.

9. Ich fühle mich bei der Nutzung der App sehr sicher.
    1.
    2.
    3.

10. Ich musste viel üben, bevor ich mit der App arbeiten konnte.
    1.
    2.

3.
