# Supplementary material for: Medical Correctness and User Friendliness of Available Apps for Cardiopulmonary Resuscitation: Systematic Search Combined With Guideline Adherence and Usability Evaluation
Source: JMIR Mhealth Uhealth. 2018 Nov 6;6(11):e190. doi: 10.2196/mhealth.9651 (PMC6246966; doi:10.2196/mhealth.9651)
Supplement: Multimedia Appendix 2 [file mhealth_v6i11e190_app2.pdf]

Multimedia Appendix 2 - Apps analysed for guideline adherence

1	Alpha First Aid
2	Audi BKK Notfall-Hilfe
3	BHF Pocket CPR
4	Cederroth Notfall App
5	CPR & Choking
6	CPR Assistant
7	Erste Hilfe Grundwissen- Weisses Kreuz
8	Erste Hilfe Hand
9	Erste-Hilfe App
10	First Aid for Cyclists
11	First Aid Plus
12	First-Aid (Erste Hilfe weisses Kreuz)
13	HALLESCHE Notfall-App
14	HAMBURG SCHOCKT
15	HELP Notfall
16	Kardiopulmonale Reanimation
17	Leben Retten
18	Livesaver Mobile
19	MediCode
20	MeinDRK – Die Rotkreuz-App des DRK e.V.
21	Notfall-App von Gelbe Seiten
22	Notfall-Hilfe (audi)
23	PocketCPR
24	Resus Algorithms
25	Richtig helfen
26	Samariterbund Österreich
27	Savealife
28	St John NZ CPR
29	St John Wales First Aid
30	St. John Ambulance First Aid
31	Standard Erste Hilfe
32	Staying Alive
33	Tiny Hearts First Aid
34	Notfall-Hilfe
